# Microglial Extracellular Vesicles as Modulators of Brain Microenvironment in Glioma

**DOI:** 10.3390/ijms232113165

**Published:** 2022-10-29

**Authors:** Myriam Catalano, Carmela Serpe, Cristina Limatola

**Affiliations:** 1 Department of Physiology and Pharmacology, Sapienza University, 00185 Rome, Italy; 2 Laboratory Affiliated to Istituto Pasteur Italia, Department of Physiology and Pharmacology, Sapienza University, 00185 Rome, Italy; 3 IRCCS Neuromed, 86077 Pozzilli, Italy

**Keywords:** extracellular vesicles, microglia, glioma, CNS, immune evasion

## Abstract

Microglial cells represent the resident immune elements of the central nervous system, where they exert constant monitoring and contribute to preserving neuronal activity and function. In the context of glioblastoma (GBM), a common type of tumor originating in the brain, microglial cells deeply modify their phenotype, lose their homeostatic functions, invade the tumoral mass and support the growth and further invasion of the tumoral cells into the surrounding brain parenchyma. These modifications are, at least in part, induced by bidirectional communication among microglial and tumoral cells through the release of soluble molecules and extracellular vesicles (EVs). EVs produced by GBM and microglial cells transfer different kinds of biological information to receiving cells, deeply modifying their phenotype and activity and could represent important diagnostic markers and therapeutic targets. Recent evidence demonstrates that in GBM, microglial-derived EVs contribute to the immune suppression of the tumor microenvironment (TME), thus favoring GBM immune escape. In this review, we report the current knowledge on EV formation, biogenesis, cargo and functions, with a focus on the effects of microglia-derived EVs in GBM. What clearly emerges from this analysis is that we are at the beginning of a full understanding of the complete picture of the biological effects of microglial-derived EVs and that further investigations using multidisciplinary approaches are necessary to validate their use in GBM diagnosis and therapy.

## 1. Introduction

Primary brain tumors are the most malignant cancers in adults [1]. Among them, malignant glioma (glioblastoma multiforme, GBM) is the most frequent (with an incidence of 9.4/100,000), with an overall patient survival of 12–15 months from diagnosis [2]. Despite an aggressive therapeutical protocol that includes surgery, radio- and chemotherapy, tumor invasion into the surrounding brain parenchyma prevents its complete eradication and is the reason for recurrence.

Microglia constitute from 5 to 20% of all glial cell populations in the central nervous system (CNS) [3] and represent the resident immune cell population of the brain, continuously scanning surrounding cells and reacting to neuronal activity and to cellular alterations. Under inflammatory conditions, microglia display an active response that reduces CNS damage and supports neuronal tissue repair. Dysregulation or the over-activation of microglial immune response is a crucial cue in nearly all CNS diseases, including GBM [4,5].

In the GBM microenvironment, the communication between microglial and tumor cells is important for tumor growth as well as for the activation of microglia-mediated protective and tumor-promoting mechanisms. Microglial and GBM cells bidirectionally communicate through direct cell-to-cell contact or via soluble released factors such as chemokines, cytokines, growth factors and matrix metalloproteinase. In addition to these soluble factors, extracellular vesicles (EVs) represent a very well-established pathway of communication in the CNS [6].

**Figure 1:**
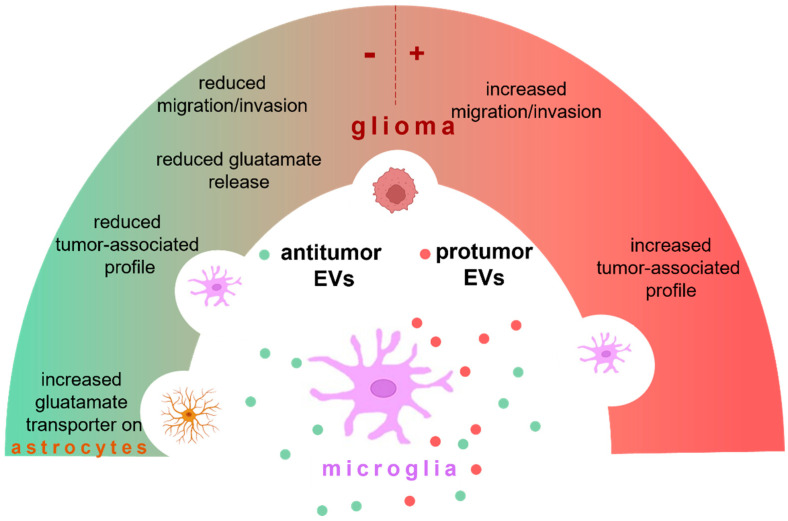
Microglia-derived EVs in glioma.

In this review, we specifically discuss the contribution of EVs released by microglia on the growth and maintenance of GBM and on anti-tumor effects. An overview of microglia EV effects in glioma is provided in Figure 1.

Microglia may produce EVs with an anti-tumor function (in green) or protumor effect (in red). These EVs target microglia, astrocytes, and glioma cells. Microglial EVs exacerbate glioma by promoting the tumor-associated profile of microglia and increasing migration and invasion of tumor cells. Otherwise, microglial EVs contrast the tumor by reducing the pro-tumoral phenotype of microglia, reducing migration and invasion of glioma cells, enhancing the expression of glutamate transporters on astrocytes, and restoring homeostasis. The cargo of microglial EVs may depend on the microglial phenotype (neuroprotective or neurotoxic) and on their biogenesis process (see details in the text).

## 2. Extracellular Vesicles

All living cells are capable of secreting EVs. The production of EVs was initially described as a mechanism for the clearance of unnecessary compounds from the cells [7]. In 1967, researchers identified small lipid vesicles derived from whole serum as well as from platelets after ultracentrifugation. This material was initially referred to as “platelet dust” and later identified as composed of microparticles. In 1971, Aaroson described the membranous structures as EVs [8]. EVs of different sizes were visualized using electron microscopy and it took a long time before the hypothesis that considered EVs as experimental “artifacts” was finally excluded. In 1981, Trams observed that shed “microvesicles” collected from the culture medium of glioblastoma cell lines had a membrane composition similar to specific plasma membrane domains and that these vesicles induced specific effects on receiving cells [9]. Despite the initial evidence, for many years, EVs have been largely overlooked. Only one decade ago, the production of EVs started to be recognized as a new possible mechanism of intercellular communication [10]. EVs have heterogeneous structures delimited by a lipid bilayer and because they lack a functional nucleus, cannot self-replicate. EVs are divided into two categories based on dimension: medium/large EVs (m/lEVs) and small EVs (sEVs). These two populations differ in size, in the mechanism of their formation and in cargo. Concerning the cargo, all EVs can transport molecules with biological activity such as proteins, lipids, and nucleic acids, including DNA and different kinds of RNAs, such as mRNA and microRNA (miRNA). miRNAs are small non-coding molecules able to bind to complementary sequences in the 3′-untranslated regions (3′UTRs) of target mRNAs, defining the post-transcriptional regulation of different genes [11].

Much has been learned on the content of EVs but the identification of cargo specific for small or medium/large vesicles is an ongoing area of intense research. 

Because of their capacity to exchange molecules between cells, EVs act as signaling structures both under physiological and pathological conditions. Specifically, in pathological conditions, EVs might act in favor of disease, such as in multiple sclerosis [12], Alzheimer’s [13,14], prion [15], and Huntington’s disease [16]. These aspects, together with the identification of m/lEVs in different body fluids, increased the interest in research to elucidate the vesicle functions in different contexts [17]. In addition, EVs transport biologically active factors across the blood–brain barrier (BBB) and the choroid plexus [18], making them potential tools for the early diagnosis of neurological disease [19] and potential vehicles for non-invasive therapies [20]. 

### 2.1. Medium/Large EVs

According to the physical characterization and to the guidelines of the International Society of Extracellular Vesicles (ISEV) [21], m/lEVs comprise all the vesicles with a diameter larger than 200 nm. They are produced by the direct invagination of the plasma membrane and then released into the extracellular space [22]. 

Each cell type changes the EVs composition according to its physiological state, regardless of its size, with peculiar lipids, proteins, and nucleic acids content [23]. The process of plasma membrane blebbing is typical of m/lEVs, even if the underlying mechanisms remain incompletely understood [23]. 

Recently, it was reported that the process involved in membrane curvatures during EV formation requires the interaction of the arrestin domain-containing protein-1 (ARRDC1) with the endosomal tumor susceptibility protein 101 (TSG101) and that this interaction leads to the transfer of TSG101 from the endosomal compartment to the plasma membrane. The presence of TSG101 in the plasma membrane allows the release of m/lEVs containing TSG101 and ARRDC1, together with other molecules. Other mechanisms are involved in the process of plasma membrane curvature. For example, the enrichment of proteins at the cellular periphery and the pressure generated by their interaction could contribute to shaping changes and curvature. This process indicates that the enrichment of protein at the site of m/lEVs budding might be a stimulus sufficient to start vesicle formation [24]. Another important factor is the alteration in the lipid composition that modifies membrane rigidity and curvature [25], key events for m/lEV formation. Phospholipids are made up of hydrocarbon tails and large head groups that give them a conical shape, with an irregular distribution. The membrane curvature is determined by the distribution of phospholipids in the plasma membrane but also by the presence of aminophospholipid translocases, such as flippases and floppases, similarly to what is described for the Golgi vesicles [26]. 

### 2.2. Small EVs

Differently, sEVs are vesicles with a diameter smaller than 200 nm [21]. At the beginning of the 1980s, a pathway that includes endocytosis was described for sEV formation. It includes the internalization of extracellular ligands and cellular components that will then be transferred again to the plasma membrane and/or degraded [27]. Upon degradation, early endosomes will be the first to be produced, followed by late endosomes [28], which accumulate intraluminal vesicles (ILVs), or multivesicular endosomes (MVBs), inside. Proteins and lipids contained in the MVBs contribute to the curvature of the early endosomal membrane. Mostly, MVBs fuse with lysosomes and their content is degraded by hydrolases. In other cases, they can fuse with clusters of the plasma membrane enriched in tetraspanin CD63, lysosomal-associated membrane proteins (LAMP1 and LAMP2) and other molecules typical of late endosomes, releasing the content into the extracellular space [29,30]. 

To exert biological effects, EVs can either be internalized or activate receptor–ligand signaling on the surface of target cells. In the last few years, the mechanisms of EVs–cell interaction have also been investigated, taking advantage of drugs or antibodies to block specific signaling pathways. These studies revealed that EVs can be internalized by target cells through different mechanisms, such as clathrin-mediated endocytosis, phagocytosis, micropinocytosis and plasma or endosomal membrane fusion [31,32,33,34,35], in addition to protein–protein interactions mediated by tetraspanins (as CD63, CD9 and CD81), integrins and immunoglobulins, proteoglycan, and lectins [36]. 

## 3. Microglial EVs: Biogenesis, Markers and Functions

Microglial-derived EVs carry lipids, proteins, DNA and RNAs to target cells, conveying different packages of information [37] according to their activation state. One function of microglia-derived EVs is to transfer cytokines to distant brain regions, regulating inflammation [38].

Since no cell-specific mechanism of EV formation has been identified so far, microglial EV biogenesis does not differ from what is described for all the other cells (see Section 2). The number of vesicles released by microglial cells can, however, be regulated: ATP-mediated activation of P2X7 receptors is involved in EV release in N9 cells and in primary microglial cells. ATP stimulation also affects EVs’ protein content, modulating the level of proteins involved in cell adhesion and phagocytosis, energy metabolism and in autophagy [39]. Furthermore, ATP stimulation specifically increases Tau protein expression in microglial EVs [40]. Microglial-derived EVs are also involved in the process of synaptic pruning, tagging synapses through the complement protein C1q [41]. 

Another stimulus for EV production by microglial cells is the protein capsaicin: the activation of its receptor, the transient receptor potential vanilloid type I (TRPVI), increases the production of m/lEVs [42]. α-synuclein (α-syn) also stimulates the production of microglia-derived EVs leading to the formation of vesicles enriched in TNF-α and in the major histocompatibility complex (MCH)-II receptor. α-syn can be also released by microglial EVs [43]. 

Microglial-derived EVs play modulatory roles in pathological conditions. In traumatic brain injuries (TBI), microglial-derived EVs propagate inflammatory signals [44]. Microglial stimulation with LPS-enriched EVs with IL1-β, TNF-α, CCL2, IL-6, NOS-2 and miR-155, thus transferring the pro-inflammatory information to the recipient cells. The proteomic analysis of sEVs derived from rat microglia isolated from the spinal cord and the cerebral cortex of control and LPS-treated animals revealed different compositions and inflammatory effects [45]. All vesicles derived from microglia express the typical exosomal markers tetraspanin CD81, Anxa2, S100, and C1q. The sEVs derived from the microglia isolated from healthy animals are enriched in proteins involved in neurodevelopment such as axonal growth promoters, neuroprotective factors, and promoters of neurites outgrowth. Instead, sEVs derived from microglia isolated from LPS-treated animals transport proteins involved in chemotaxis, in the activation of the NFkB pathway, metabolic processes, and transport pro-inflammatory chemokines and cytokines such as IL-6 and TNF-α [45].

Other regulators of microglial EV release are neurotransmitters. For example, serotonin (5-HT) induces EV release from microglia through the stimulation of 5-HTR2 and 5-HTR4. The activation of 5-HTRs increases the intracellular levels of Ca^2+^ and cAMP, both enhancing EV secretion. Even if a direct observation is lacking, authors postulate a specific effect of microglia-derived EVs on serotoninergic neurons [46], widening the mechanisms of communication between neurons and microglia. Another stimulus affecting the protein composition of microglial EVs is Wnt3a. Wnt3a stimulation in fact enriched EVs with proteins associated with cellular metabolism, cell architecture and protein synthesis [47], as well as ubiquitin and proteasome components (proteasome subunit B type 7 and type 2), suggesting a role of microglia EVs in the clearance of the extracellular space [47].

A summary of the factors modulating microglial EVs is reported in Table 1.

In addition to metabolic enzymes, chaperones and tetraspanins, microglia-derived EVs also carry membrane receptors and specific cellular markers such as CD13 and MCT-1 [48]. The lipid composition of EVs changes with their dimensions and affects their content. The most abundant lipids present in EVs are cholesterol (CHOL), sphingomyelin (SM), glycol sphingolipids and phosphatidylserine (PS). sEVs are enriched in CHOL and SM [49]. Even if there is no precise evidence for a specific functional role of lipids in microglia-derived EVs, one study demonstrates that the lipid fraction of microglial EVs affects the maturation of oligodendrocyte precursor cells (OPCs) [50]. In particular, sphingosine-1-phosphate (S1P) acts as an attractive guide for OPC migration to myelin lesion sites [50]. Furthermore, lipids in microglia-derived EVs recognize and target different recipient cells [47]. Another lipid enriched in microglia-derived EVs is N-arachidonoylethanolamine (N-AEA), which modulates synaptic transmission. N-AEA is enriched in EVs derived both from N9 cells and primary rat microglia [51].

Different studies highlight the role of EVs in miRNA transfer in the brain [52,53]. The miRNA content in microglia-derived EVs revealed a two-fold enrichment of miR-1860, miR-1705, miR-2284y-6, miR-146a, miR-858, and miR-7718 [54]. Different miRNAs are present in EVs according to different activation states of microglial cells [52]. For example, miR-146-5p, miR-181a and miR-223 are increased upon LPS treatment [52]. These miRNAs have synaptic targets: for example, miR-146 downregulates synaptotagmin 1 (Syt1) and neuroligin 1 (Nlg1) levels in neurons, with a reduction of dendritic spines and synaptic density [52]. In addition, EVs derived from IL-4-stimulated BV2 cells are enriched in miR-26a that promotes angiogenesis [55], revealing another microglial EV-regulated cellular function.

Microglial-derived EVs contribute to maintaining microglia homeostasis influencing their phenotype and gene expression profile. Several studies were conducted to elucidate specific functions regulated by EVs derived from microglial cells under different states of activation, as stated in the previous section. Regardless of the status of the donor cells, microglial EVs modulate, in the target cells, the expression of crucial genes that regulate molecular intracellular pathways such as neuroinflammation (by activating the multimers of the inflammasome), apoptosis and autophagy [56].

As one example, during cellular stress, microglial EVs, acting on other microglial cells, increase the expression of the microtubule-associated protein 1A/1B light chain 3B isoform II (LC3B-II), which is an autophagic marker [56].

Microglia-derived EVs were also investigated for their contribution to neuroprotection. Neuroprotection is defined as the ability to avoid neuronal cell death by inhibiting the signaling pathways activated by cell dysfunction and cell death under pathological conditions. Microglia-derived EVs affect neurite outgrowth in rat primary neurons, due to the transfer of six different miRNAs that are able to regulate the physiological regenerative process in neurons [56].

## 4. Microglial EVs in Brain Tumors

In GBM, microglia and infiltrating macrophages represent about 30% of total cells in the tumor mass, contributing to the early anti-tumor immune response and later playing a role in supporting cancer growth [57,58]. In fact, brain tumor cells attract microglia by secreting factors such as cytokines, growth factors, chemokines and colony-stimulating factors [58], which induce phenotypical and genetical switch of microglia toward a pro-tumorigenic ally. Microglia and infiltrating macrophages, modified by cancer cells, are defined as tumor-associated myeloid cells (TAMCs) [59].

In addition to secreted molecules, microglia-tumor communication is also mediated by gap junctions, tunneling nanotubes and EVs [60]. EVs make possible communication along distant sites and, importantly, also permit bidirectional cell-to-cell communication [60]. As stated before, EVs can deliver not only soluble proteins but also a wide variety of coding and non-coding RNAs that can alter the gene expression of the target cells [60].

## 5. EV-Mediated Communication between GBM and the Brain Tumor Environment

GBM is the most aggressive brain tumor and represents an important research and medical challenge [1]. EVs released by GBM cells have specific cargos that favor tumor propagation. They contain oncogenes, such as the epidermal growth factor receptor (EGFRvIII), that induce the expression of other oncogenes (i.e., p27 and Bcl-xL) and the activation of pro-tumoral pathways (i.e., AKT and ERK1/2 phosphorylation) [61]. Another mechanism of EV-mediated progression of glioma is the transfer of the RNA-binding motif 11 (RBM11) [62], a pro-tumoral protein that promotes invasion and proliferation [63] by apoptotic glioma cells. Additionally, EVs derived from glioma stem cells (GSCs) contribute to maintaining the GBM cellular heterogeneity (a peculiarity of high malignancy) [64]. In addition to the horizontal propagation of the oncogenic activity, glioma-released EVs also convey materials to non-tumoral cells [65], such as astrocytes, neurons, and endothelial and immune cells.

Astrocytes stimulated by GBM-derived EVs acquire a tumor-supportive phenotype [6]. In addition, GBM-derived EVs enhance the proliferation and the transformation of astrocytes by RNAs able to reprogram metabolic activity [66]. Conversely, exosome-mediated transport of miR-19a from astrocytes to tumor cells critically results in PTEN downregulation and tumor growth [67,68].

GBM-derived EVs can regulate neuronal excitability [69] and miRs transfer (miR-148a and miR-9-5p) promotes angiogenesis in endothelial cells [70,71,72,73]. In bidirectional communication, brain endothelial cells release EVs containing tetraspanin CD9 to GBM [74], enhancing tumor progression through the inhibition of ubiquitination of IL6 receptor gp30 and promoting the activation of the signal transducer and activator of transcription 3 (STAT3) [75,76,77,78]. Endothelial-derived EVs overexpressing the tumor suppressor esophageal cancer-related gene-4 (ECRG4) inhibit glioma cell proliferation [79].

GBM-derived EVs suppress the activation of T lymphocytes, partially through the programmed death ligand-1 (PD-L1) [80], a key player of the tumor immune escape in many cancers including GBM [81,82]. Macrophages stimulated with GBM-derived EVs acquire a pro-tumoral phenotype (i.e., overexpression of Arg-1 and IL-10 and downregulation of iNOS and TNF-α), in part through miR-10b-5p [83]. Furthermore, GBM-released EVs also block the clonal proliferation of T cells by transferring CD73 [84], which inhibits aerobic glycolysis [85].

GBM-derived EVs are involved in the initiation of the tumor-supportive TAMC phenotype [86]. TAMCs efficiently engulf EVs, as visualized in vivo, in glioma-bearing mice [87]. GBM-derived EVs increase the phagocytic activity of microglia towards the extracellular matrix, creating free space in the brain parenchyma [88]. One of the mechanisms involved is the transfer of the membrane type 1-matrix metalloproteinase (MT1-MMP) [88], which degrades the proteins of the extracellular matrix. GBM-derived EVs affect TAMC proliferation both in vivo and in vitro [89]. GBM-derived EVs contain miR-21 [90] which downregulates the target tumor-suppressive gene Btg2 (B cell translocation gene 2) [91] in microglial cells. Moreover, they transfer the Wilms tumor-1 (WT1) protein to microglial cells, upregulating the expression of thrombospondin-1 (Thbs-1), a key promoter of angiogenesis [92]. GSCs represent a cell subpopulation relevant for the tumor resistance to radiations and release EVs able to polarize microglial cells towards a pro-tumoral phenotype, specifically by transferring miR-504 that inhibits the putative onco-suppressor gene grb10 (growth factor receptor-bound protein 10) [90]. miR-504 also increases the pro-tumoral markers CD209 and TGF-β and decreases the expression of the anti-tumoral marker genes CD86 and TNF-α [90]. GBM-released EVs reprogram microglia towards a pro-tumoral phenotype also through the long noncoding RNA (lncRNA) associated with temozolomide (TMZ) (lnc-TALC). lnc-TALC competes with miR-20b-3p by inducing the expression of Stat3, which, in turn, activates the expression of the DNA repair enzyme O^6^-methylguanine-DNA methyltransferase (MGMT) [93]. lnc-TALC also increases Arg-1, CD163, TGFβ, IL4, and IL10 [94], enhancing the pro-tumoral phenotype of microglia.

On the other side, several pieces of evidence demonstrate that microglial-released EVs can affect the TME and the effects induced by microglial EVs vary with the activation state of these cells [95]. Specifically, m/lEVs released by LPS/INFγ-activated microglia contain transcripts for several inflammation-related genes that reduce the pro-tumoral efficacy of TAMCs. In contrast, m/lEVs released by IL4-stimulated microglia enhance the pro-tumoral phenotype of TAMCs [95]. We mentioned before that the biogenesis process and the cargo differ in sEVs and m/lEVs. In fact, in contrast with m/lEVs, sEVs released by both LPS/INFγ and IL4-stimulated microglial cells exert anti-tumoral effects in a mouse model of glioma, reducing tumor mass and prolonging mice survival [96]. Similarly, sEVs released by unstimulated microglial cells reduce the invasion of glioma cells in 3D-spheroid cultures [97]. The anti-glioma effect of microglial-derived sEVs can be mediated by miR124. This specific miR enhances the expression of the glutamate transporter GLT1 on astrocytes, with increased clearance of glutamate in the synaptic cleft. Glioma cells are in fact able to release neurotoxic amounts of glutamate that promote neuronal death and permit the invasion of tumor cells [96]. 

EV-mediated delivery of miRNAs could represent a promising approach to contrast the growth of brain tumors. In a 3D-microfluidic GBM microenvironment, miR-124-loaded EVs reduce tumor growth and inhibit microglia polarization towards a pro-tumoral phenotype [98]. Furthermore, microglia EVs can be engineered to deliver drugs to glioma cells, taking advantage of their ability to target the brain tumor mass [99]. Specifically, microglia cells were engineered to release EVs enriched in paclitaxel (PTX), a pro-apoptotic compound that blocks the cell cycle at the G2/M phase [100]. Loading PTX in EVs overcomes its low BBB permeability [101] and could be used for other drugs with a reduced capability to enter brain parenchyma.

A summary of pro-tumorigenic and anti-tumorigenic factors transported via EVs in glioma is reported in Table 2.

## 6. Conclusions 

Microglial-released EVs are active players in maintaining or restoring CNS homeostasis (Figure 1). They can be heterogeneous depending on the state of activation of microglial donor cells and, in turn, depending on the microenvironment stimuli. In addition, EVs have stable structures and high stability in the extracellular space. All these features make microglial EVs potential tools to deliver therapeutics in the CNS with the purpose of interfering with the growth and spread of tumors in the brain parenchyma. 

However, the current knowledge on the effects of EVs in the context of brain tumors is not sufficient to propose their immediate use in clinics and the data summarized in this review suggest that a more detailed description of the content of EVs in the different experimental conditions and in patients, and a better understanding of how the formation process affects their cargo, is necessary to move forward in EV-based therapeutic applications. In particular, engineered EVs could enhance the efficacy of EV-mediated anti-tumorigenic pathways. Finally, we conclude that the knowledge of central and peripheral EV content in the glioma landscape could contribute to an early diagnosis and, possibly, to future personalized therapies.

## Figures and Tables

**Table 1:** Modulating factors, specific receptors, and effects on microglial EVs.

Factor	Receptor	Effect on Microglial EVs	Reference
ATP	P2X7	increased production	[39]
		enrichment in proteins regulating cell adhesion, phagocytosis, energy metabolism, autophagy	[39]
		enrichment in Tau protein	[40]
		enrichment in C1q	[41]
capsaicin	TRPVI	increased production	[42]
α-syn		increased production	[43]
		enrichment in TNF-α and (MCH)-II receptor	[43]
LPS		enrichment in pro-inflammatory chemokines, cytokines and miRs	[45]
		enrichment in proteins regulating chemotaxis	[45]
		enrichment in proteins activating the NFkB pathway	[45]
		enrichment in proteins regulating the metabolism	[45]
5-HT	5-HTR2, 5HTR4	increased production	[46]
Wnt3		enrichment in proteins regulating cellular metabolism, cell architecture and protein synthesis	[47]
		enrichment in ubiquitin, proteasome subunit B type 7 and type 2	[47]

**Table 2:** Pro- and anti-tumorigenic factors transported by EVs in glioma microenvironment. Donor cells are listed in columns and target cells in rows.

**PRO-** **TUMOR EVs**	**GBM**	**Microglia/** **Macrophages**	**Astrocytes**	**Endothelial Cells**	**T Cells**
**GBM**	EGFRvIII [61] RBM11 [62]	miR-10b-5p [83] MT1-MMP [88] miR-21 [90] WT1 [92] miR-504 [90] lnc-TALC [93,94]	RNAs for metabolic activity [66]	miR-148a [70,71,72,73] miR-9-5p [70,71,72,73]	PD-L1 [80] CD73 [84]
**Astrocytes**	miR-19a [67,68]				
**Endothelial cells**	CD9 [74,75,76,77,78]				
**ANTI-** **TUMOR EVs**	**GBM**	**Microglia/** **macrophages**	**Astrocyte**		
**Endothelial cells**	ECRG4 [79]				
**Microglia/macrophages**	miR124 [96,98]	inflammation-related genes [95] miR124 [98]	miR124 [96]		

## Abbreviations

3′UTR	3′-untranslated regions
5-HT	serotonin
ARRDC1	arrestin domain-containing protein-1
BBB	blood–brain barrier
CHOL	cholesterol
CNS	Central Nervous System
ECRG4	tumor suppressor esophageal cancer-related gene-4
EGFRvIII	epidermal growth factor receptor
EVs	Extracellular vesicles
GBM	Glioblastoma
grb10	growth factor receptor-bound protein 10 gene
GSCs	glioma stem cells
ILVs	intraluminal vesicles
ISEV	International Society of Extracellular Vesicles
LAMP1–LAMP2	lysosomal-associated membrane proteins 1–2
LC3B-II	microtubule-associated protein 1A/1B light chain 3B isoform II
lncRNA	long noncoding RNA
lnc-TALC	long noncoding RNA (lncRNA) associated with temozolomide (TMZ)
m/lEVs	medium/large extracellular vesicles
miRNA	microRNA
MT1-MMP	membrane type 1-matrix metalloproteinase
MVBs	multivesicular endosomes
N-AEA	N-arachidonoylethanolamine
OPCs	oligodendrocyte precursor cells
PD-L1	binding of programmed death ligand-1
PS	phosphatidylserine
PTX	paclitaxel
RBM11	RNA-binding motif 11
S1P	sphingosine-1-phosphate
sEVs	small extracellular vesicles
SM	sphingomyelin
STAT3	activator of transcription 3
TAMCs	tumor-associated myeloid cells
Thbs-1	thrombospondin-1
TME	tumor microenvironment
TMZ	temozolomide
TRPVI	vanilloid type I
TSG101	protein tumor susceptibility protein 101
WT1	Wilms tumor-1
α -syn	α-synuclein

## References

[1] Ostrom Q.T., Cioffi G. (2020). CBTRUS statistical report: Primary brain and other central nervous system tumors diagnosed in the United States in 2013–2017. Neuro-Oncology.

[2] Abbruzzese C., Padey B. (2017). Drug repurposing for the treatment of glioblastoma multiforme. JECCR.

[3] Pelvig D.P., Pakkenberg H., Stark A.K., Pakkenberg B. (2008). Neocortical glial cell numbers in human brains. Neurobiol. Aging.

[4] Ginhoux F., Lim S., Hoeffel G., Low D., Huber T. (2013). Origin and differentiation of microglia. Front. Cell Neurosci..

[5] Thion M.S., Ginhoux F., Garel S. (2018). Microglia and early brain development: An intimate journey. Science.

[6] Hallal S., Mallawaaratchy D.M. (2019). Extracellular vesicles released by glioblastoma cells stimulate normal astrocytes to acquire a tumor-supportive phenotype via p53 and MYC signaling pathways. Mol. Neurobiol..

[7] Johnstone R.M., Adam M. (1987). Vesicle formation during reticulocyte maturation. Association of plasma membrane activities with released vesicles (exosomes). J. Biol. Chem..

[8] Aaronson S., Behrens U. (1971). Ultrastructure of intracellular and extracellular vesicles, membranes, and myelin figures produced by Ochromonas danica. J. Ultrastruct. Res..

[9] Trams E.G., Lauter C. (1981). Exfoliation of membrane ecto-enzymes in the form of micro-vesicles. Biochim. Biophys. Acta.

[10] Iraci N., Gaude E., Leonardi T., Costa A., Cossetti C., Peruzzotti-Jametti L., Bernstock J.D., Saini H.K., Gelati M., Vescovi A.L. (2017). Extracellular vesicles are independent metabolic units with asparaginase activity. Nat. Chem. Biol..

[11] de Sousa M.C., Gjorgjieva M., Dolicka D., Sobolewski C., Foti M. (2019). Deciphering miRNAs’ Action through miRNA Editing. Int. J. Mol. Sci..

[12] Carandini T., Colombo F., Finardi A., Casella G., Garzetti L., Verderio C., Furlan R. (2015). Microvesicles: What is the Role in Multiple Sclerosis?. Front. Neurol..

[13] Aguzzi A., Rajendran L. (2009). The Transcellular Spread of Cytosolic Amyloids, Prions, and Prionoids. Neuron.

[14] Gouwens N.W., Berg J., Feng D., Sorensen S.A., Zeng H., Hawrylycz M.J., Koch C., Arkhipov A. (2018). Systematic generation of biophysically detailed models for diverse cortical neuron types. Nat. Commun..

[15] Fevrier B., Vilette D., Archer F., Loew D., Faigle W., Vidal M., Laude H., Raposo G. (2004). Cells release prions in association with exosomes. Proc. Natl. Acad. Sci. USA.

[16] Zhang X., Abels E.R., Redzic J.S., Margulis J., Finkbeiner S., Breakefield X.O. (2016). Potential Transfer of Polyglutamine and CAG-Repeat RNA in Extracellular Vesicles in Huntington’s Disease: Background and Evaluation in Cell Culture. Cell. Mol. Neurobiol..

[17] Fleissner F., Goerzig Y. (2012). Microvesicles as novel biomarkers and therapeutic targets in transplantation medicine. Am. J. Transplant..

[18] Balusu S., Van Wonterghem E., De Rycke R., Raemdonck K., Stremersch S., Gevaert K., Brkic M., Demeestere D., Vanhooren V., Hendrix A. (2016). Identification of a novel mechanism of blood–brain communication during peripheral inflammation via choroid plexus-derived extracellular vesicles. EMBO Mol. Med..

[19] Fiandaca M.S., Kapogiannis D., Mapstone M., Boxer A., Eitan E., Schwartz J.B., Abner E.L., Petersen R.C., Federoff H.J., Miller B.L. (2015). Identification of preclinical Alzheimer’s disease by a profile of pathogenic proteins in neurally derived blood exosomes: A case-control study. Alzheimer’s Dement..

[20] Alvarez-Erviti L., Seow Y. (2011). Delivery of siRNA to the mouse brain by systemic injection of targeted exosomes. Nat. Biotechnol..

[21] Théry C., Witwer K.W., Aikawa E., Alcaraz M.J., Anderson J.D., Andriantsitohaina R., Antoniou A., Arab T., Archer F., Atkin-Smith G.K. (2018). Minimal information for studies of extracellular vesicles 2018 (MISEV2018): A position statement of the International Society for Extracellular Vesicles and update of the MISEV2014 guidelines. J. Extracell. Vesicles.

[22] Tricarico C., Clancy J., D’Souza-Schorey C. (2017). Biology and biogenesis of shed microvesicles. Small GTPases.

[23] Colombo M., Raposo G., Théry C. (2014). Biogenesis, secretion, and intercellular interactions of exosomes and other extracellular vesicles. Annu. Rev. Cell Dev. Biol..

[24] Stachowiak J.C., Schmid E.M., Ryan C.J., Ann H.S., Sasaki D.Y., Sherman M.B., Geissler P.L., Fletcher D.A., Hayden C.C. (2012). Membrane bending by protein–protein crowding. Nat. Cell Biol..

[25] Stachowiak J.C., Brodsky F., Miller E. (2013). A cost–benefit analysis of the physical mechanisms of membrane curvature. Nat. Cell Biol..

[26] Karaeva A.R., Khaskov M.A., Mitberg E.B., Kulnitskiy B.A., Perezhogin I.A., Ivanov L.A., Denisov V.N., Kirichenko A.N., Mordkovich V.Z. (2012). Longer Carbon Nanotubes by Controlled Catalytic Growth in the Presence of Water Vapor, Fullerenes, Nanotub. Carbon Nanostruct..

[27] Gould G.W., Lippincott-Schwartz J. (2009). New roles for endosomes: From vesicular carriers to multi-purpose platforms. Nat. Rev. Mol. Cell Biol..

[28] Stoorvogel W., Strous G.J., Geuze H.J., Oorschot V., Schwartzt A.L. (1991). Late endosomes derive from early endosomes by maturation. Cell.

[29] Jaiswal J., Andrews N., Simon S.M. (2002). Membrane proximal lysosomes are the major vesicles responsible for calcium-dependent exocytosis in nonsecretory cells. J. Cell Biol..

[30] Raposo G., Nijman H.W. (1996). B lymphocytes secrete antigen-presenting vesicles. J. Exp. Med..

[31] Calvo V., Izquierdo M. (2020). Inducible Polarized Secretion of Exosomes in T and B Lymphocytes. Int. J. Mol. Sci..

[32] Christianson H.C., Svensson K.J. (2013). Cancer cell exosomes depend on cell-surface heparan sulfate proteoglycans for their in-ternalization and functional activity. Proc. Natl. Acad. Sci. USA.

[33] Svensson K.J., Christianson H.C. (2013). Exosome uptake depends on ERK1/2-heat shock protein 27 signalling and lipid raft-mediated endocytosis negatively regulated by caveolin-1. J. Biol. Chem..

[34] Morelli A.E., Larregina A.T. (2004). Endocytosis, intracellular sorting, and processing of exosomes by dendritic cells. Blood.

[35] Tumne A., Prasad V.S. (2009). Noncytotoxic suppression of human immunode- ficiency virus type 1 transcription by exosomes se-creted from CD^8^^+^ T cells. J. Virol..

[36] Thali M. (2009). The Roles of Tetraspanins in HIV-1 Replication. Curr. Top. Microbiol. Immunol..

[37] Ceccarelli L., Giacomelli C., Marchetti L., Martini C. (2021). Microglia extracellular vesicles: Focus on molecular composition and biological function. Biochem. Soc. Trans..

[38] Fruhbeis C., Frohlich D. (2013). Extracellular vesicles as mediators of neuron-glia communication. Front. Cell. Neurosci..

[39] Drago F., Lombardi M. (2017). ATP modifies the proteome of extracellular vesicles released by microglia and influences their action on astrocytes. Front. Pharmacol..

[40] Daniele S., Giacomelli C. (2018). Brain ageing and neurodegenerative disease: The role of cellular waste management. Biochem. Pharmacol..

[41] Stevens B., Allen N.J. (2007). The classical complement cascade mediates CNS synapse elimination. Cell.

[42] Marrone M.C., Morabito A., Giustizieri M., Chiurchiù V., Leuti A., Mattioli M., Marinelli S., Riganti L., Lombardi M., Murana E. (2017). TRPV1 channels are critical brain inflammation detectors and neuropathic pain biomarkers in mice. Nat. Commun..

[43] Chang C., Lang H. (2013). Exosomes of BV-2 cells induced by alpha-synuclein: Important mediator of neurodegeneration in PD. Neurosci. Lett..

[44] Kumar A.A., Stoica B.A. (2017). Microglial-derived microparticles mediate neuroinflammation after traumatic brain injury. J. Neuroinflamm..

[45] Yang Y., Boza-Serrano A. (2018). Inflammation leads to distinct populations of extracellular vesicles from microglia. J. Neuroinflamm..

[46] Glebov K., Löchner M. (2015). Serotonin stimulates secretion of exosomes from microglia cells. Glia.

[47] Hooper C., Sainz-Fuertes R. (2012). Wnt3a induces exosome secretion from primary cultured rat microglia. BMC Neurosci..

[48] Potolicchio I., Carven G.J. (2005). Proteomic analysis of microglia-derived exosomes: Metabolic role of the aminopeptidase CD13 in neuropeptide catabolism. J. Immunol..

[49] Pienimaeki-Roemer A., Kuhlmann K. (2015). Lipidomic and proteomic characterization of platelet extracellular vesicle subfractions from senescent platelets. Transfusion.

[50] Lombardi M., Parolisi R., Scaroni F., Bonfanti E., Gualerzi A., Gabrielli M., de Rosbo N.K., Uccelli A., Giussani P., Viani P. (2019). Detrimental and protective action of microglial extracellular vesicles on myelin lesions: Astrocyte involvement in remyelination failure. Acta Neuropathol..

[51] Gabrielli M., Battista N. (2015). Active endocannabinoids are secreted on extracellular membrane vesicles. EMBO Rep..

[52] Prada I., Gabrielli M., Turola E., Iorio A., D’Arrigo G., Parolisi R., De Luca M., Pacifici M., Bastoni M., Lombardi M. (2018). Glia-to-neuron transfer of miRNAs via extracellular vesicles: A new mechanism underlying inflammation-induced synaptic alterations. Acta Neuropathol..

[53] Raffo-Romero A., Arab T. (2018). Medicinal leech CNS as a model for exosome studies in the crosstalk between microglia and neurons. Int. J. Mol. Sci..

[54] Broek B.V.D., Pintelon I., Hamad I., Kessels S., Haidar M., Hellings N., Hendriks J.J., Kleinewietfeld M., Brône B., Timmerman V. (2020). Microglial derived extracellular vesicles activate autophagy and mediate multi-target signaling to maintain cellular homeostasis. J. Extracell. Vesicles.

[55] Tian Y., Zhu P., Liu S., Jin Z., Li D., Zhao H., Zhu X., Shu C., Yan D., Dong Z. (2019). IL-4-polarized BV2 microglia cells promote angiogenesis by secreting exosomes. Adv. Clin. Exp. Med..

[56] Lemaire Q., Raffo-Romero A. (2019). Isolation of microglia-derived extracellular vesicles: Towards miRNA signatures and neu-ro-protection. J. Nanobiotechnol..

[57] Borst K., Dumas A.A., Prinz M. (2021). Microglia: Immune and non-immune functions. Immunity.

[58] Graeber M.B., Scheithauer B.W., Kreutzberg G.W. (2002). Microglia in brain tumors. Glia.

[59] Hambardzumyan D., Gutmann D.H., Kettenmann H. (2016). The role of microglia and macrophages in glioma maintenance and progression. Nat. Neurosci..

[60] Skog J., Würdinger T. (2008). Glioblastoma microvesicles transport RNA and proteins that promote tumour growth and provide diagnostic biomarkers. Nat. Cell Biol..

[61] Al-Nedawi K., Meehan B., Micallef J., Lhotak V., May L., Guha A., Rak J. (2008). Intercellular transfer of the oncogenic receptor EGFRvIII by microvesicles derived from tumour cells. Nat. Cell Biol..

[62] Pavlyukov M.S., Yu H., Bastola S., Minata M., Shender V.O., Lee Y., Zhang S., Wang J., Komarova S., Wang J. (2018). Apoptotic Cell-Derived Extracellular Vesicles Promote Malignancy of Glioblastoma Via Intercellular Transfer of Splicing Factors. Cancer Cell.

[63] Fu C., Yuan M., Sun J., Liu G., Zhao X., Chang W., Ma Z. (2021). RNA-Binding Motif Protein 11 (RBM11) Serves as a Prognostic Biomarker and Promotes Ovarian Cancer Progression. Dis. Markers.

[64] Ricklefs F., Mineo M., Rooj A.K., Nakano I., Charest A., Weissleder R., Breakefield X.O., Chiocca E.A., Godlewski J., Bronisz A. (2016). Extracellular Vesicles from High-Grade Glioma Exchange Diverse Pro-oncogenic Signals That Maintain Intratumoral Heterogeneity. Cancer Res..

[65] Gao X., Zhang Z., Mashimo T., Shen B., Nyagilo J., Wang H., Wang Y., Liu Z., Mulgaonkar A., Hu X.-L. (2020). Gliomas Interact with Non-glioma Brain Cells via Extracellular Vesicles. Cell Rep..

[66] Zeng A., Wei Z., Rabinovsky R., Jun H.J., El Fatimy R., Deforzh E., Arora R., Yao Y., Yao S., Yan W. (2020). Glioblastoma-Derived Extracellular Vesicles Facilitate Transformation of Astrocytes via Reprogramming Oncogenic Metabolism. iScience.

[67] Brandao M., Simon T., Critchley G., Giamas G. (2019). Astrocytes, the rising stars of the glioblastoma microenvironment. Glia.

[68] Zhang L., Zhang S., Yao J., Lowery F.J., Zhang Q., Huang W.-C., Li P., Li M., Wang X., Zhang C. (2015). Microenvironment-induced PTEN loss by exosomal microRNA primes brain metastasis outgrowth. Nature.

[69] Spelat R., Jihua N., Triviño C.A.S., Pifferi S., Pozzi D., Manzati M., Mortal S., Schiavo I., Spada F., Zanchetta M.E. (2022). The dual action of glioma-derived exosomes on neuronal activity: Synchronization and disruption of synchrony. Cell Death Dis..

[70] Lucero R., Zappulli V., Sammarco A., Murillo O.D., Cheah P.S., Srinivasan S., Tai E., Ting D.T., Wei Z., Roth M.E. (2020). Glioma-Derived miRNA-Containing Extracellular Vesicles Induce Angiogenesis by Reprogramming Brain Endothelial Cells. Cell Rep..

[71] Kim J., Zhang Y. (2014). MicroRNA-148a is a prognostic oncomiR that targets MIG6 and BIM to regulate EGFR and apoptosis in glioblastoma. Cancer Res..

[72] Chen X., Yang F. (2019). MiR-9 promotes tumorigenesis and angiogenesis and is activated by MYC and OCT4 in human glioma. J. Exp. Clin. Cancer Res..

[73] Wong H.A., Fatimy R.E. (2015). The Cancer Genome Atlas analysis predicts microRNA for targeting cancer growth and vascularization in glioblastoma. Mol. Ther..

[74] Podergajs N., Motaln H., Rajčević U., Verbovšek U., Koršič M., Obad N., Espedal H., Vittori M., Herold-Mende C., Miletic H. (2016). Transmembrane protein CD9 is glioblastoma biomarker, relevant for maintenance of glioblastoma stem cells. Oncotarget.

[75] Li D., Tian Y., Hu Y., Qi Y., Tian N., Li S., Hu P., Wu F., Wei Q., Wei Z. (2019). Glioma-associated human endothelial cell-derived extracellular vesicles specifically promote the tumourigenicity of glioma stem cells via CD9. Oncogene.

[76] Shi Y., Zhou W. (2017). Tetraspanin CD9 stabilizes gp130 by preventing its ubiquitin-dependent lysosomal degradation to promote STAT3 activation in glioma stem cells. Cell Death Differ..

[77] Ouédraogo Z.G., Biau J. (2017). Role of STAT3 in genesis and progression of human malignant gliomas. Mol. Neurobiol..

[78] Chang N., Ahn S.H. (2017). The role of STAT3 in glioblastoma progression through dual influences on tumor cells and the immune microenvironment. Mol. Cell. Endocrinol..

[79] Huo H., Yang S., Wu H., Sun Y., Zhao R., Ye R., Yan D., Shi X., Yang J. (2021). Brain endothelial cells-derived extracellular vesicles overexpressing ECRG4 inhibit glioma proliferation through suppressing inflammation and angiogenesis. J. Tissue Eng. Regen. Med..

[80] Ricklefs F.L., Alayo Q., Krenzlin H., Mahmoud A.B., Speranza M.C., Nakashima H., Hayes J.L., Lee K., Balaj L., Passaro C. (2018). Immune evasion mediated by PD-L1 on glioblastoma-derived extracellular vesicles. Sci. Adv..

[81] Keir M.E., Butte M.J., Freeman G.J., Sharpe A.H. (2008). PD-1 and Its Ligands in Tolerance and Immunity. Annu. Rev. Immunol..

[82] Wang H., Xiao Y., Ren X., Wan D. (2021). Prognostic value of programmed death ligand 1 (PD-L1) in glioblastoma: A systematic review, meta-analysis and validation based on dataset. Bioengineered.

[83] Li B., Yang C., Zhu Z., Chen H., Qi B. (2022). Hypoxic glioma-derived extracellular vesicles harboring MicroRNA-10b-5p enhance M2 polarization of macrophages to promote the development of glioma. CNS Neurosci. Ther..

[84] Azambuja J.H., Gelsleichter N.E., Beckenkamp L.R., Iser I.C., Fernandes M.C., Figueiró F., Battastini A.M.O., Scholl J.N., de Oliveira F.H., Spanevello R.M. (2019). CD73 Downregulation Decreases In Vitro and In Vivo Glioblastoma Growth. Mol. Neurobiol..

[85] Bavaresco L., Bernardi A., Braganhol E., Cappellari A.R., Rockenbach L., Farias P.F., Wink M.R., Delgado-Cañedo A., Battastini A.M.O. (2008). The role of ecto-5′-nucleotidase/CD73 in glioma cell line proliferation. Mol. Cell. Biochem..

[86] de Vrij J., Maas S.N., Kwappenberg K.M., Schnoor R., Kleijn A., Dekker L., Luider T.M., de Witte L.D., Litjens M., van Strien M.E. (2015). Glioblastoma-derived extracellular vesicles modify the phenotype of monocytic cells. Int. J. Cancer.

[87] Van der Vos K.E., Abels E.R., Zhang X., Lai C., Carrizosa E., Oakley D., Prabhakar S., Mardini O., Crommentuijn M.H.W., Skog J. (2016). Directly visualized glioblastoma-derived extracellular vesicles transfer RNA to microglia/macrophages in the brain. Neuro-Oncology.

[88] Vinnakota K., Hu F. (2013). Toll-like receptor 2 mediates microglia/brain macrophage MT1-MMP expression and glioma expan-sion. Neuro-Oncology.

[89] Abels E.R., Broekman M.L., Breakefield X.O., Maas S.L. (2019). Glioma EVs Contribute to Immune Privilege in the Brain. Trends Cancer.

[90] Bier A., Hong X., Cazacu S., Goldstein H., Rand D., Xiang C., Jiang W., Ben-Asher H.W., Attia M., Brodie A. (2020). miR-504 modulates the stemness and mesenchymal transition of glioma stem cells and their interaction with microglia via delivery by extracellular vesicles. Cell Death Dis..

[91] Mao B., Zhang Z., Wang G. (2015). BTG2: A rising star of tumor suppressors (Review). Int. J. Oncol..

[92] Tsutsui T., Kawahara H., Kimura R., Dong Y., Jiapaer S., Sabit H., Zhang J., Yoshida T., Nakada M., Hanayama R. (2020). Glioma-derived extracellular vesicles promote tumor progression by conveying WT1. Carcinogenesis.

[93] Wu P., Cai J., Chen Q., Han B., Meng X., Li Y., Li Z., Wang R., Lin L., Duan C. (2019). Lnc-TALC promotes O6-methylguanine-DNA methyltransferase expression via regulating the c-Met pathway by competitively binding with miR-20b-3p. Nat. Commun..

[94] Li Z., Meng X., Wu P., Zha C., Han B., Li L., Sun N., Qi T., Qin J., Zhang Y. (2021). Glioblastoma Cell–Derived lncRNA-Containing Exosomes Induce Microglia to Produce Complement C5, Promoting Chemotherapy Resistance. Cancer Immunol. Res..

[95] Grimaldi A., Serpe C., Chece G., Nigro V., Sarra A., Ruzicka B., Relucenti M., Familiari G., Ruocco G., Pascucci G.R. (2019). Microglia-Derived Microvesicles Affect Microglia Phenotype in Glioma. Front. Cell. Neurosci..

[96] Serpe C., Monaco L., Relucenti M., Iovino L., Familiari P., Scavizzi F., Raspa M., Familiari G., Civiero L., D’Agnano I. (2021). Microglia-Derived Small Extracellular Vesicles Reduce Glioma Growth by Modifying Tumor Cell Metabolism and Enhancing Glutamate Clearance through miR-124. Cells.

[97] Murgoci A.-N., Cizkova D., Majerova P., Petrovova E., Medvecky L., Fournier I., Salzet M. (2018). Brain-Cortex Microglia-Derived Exosomes: Nanoparticles for Glioma Therapy. ChemPhysChem.

[98] Hong S., You J.Y., Paek K., Park J., Kang S.J., Han E.H., Choi N., Chung S., Rhee W.J., Kim J.A. (2021). Inhibition of tumor progression and M2 microglial polarization by extracellular vesicle-mediated microRNA-124 in a 3D microfluidic glioblastoma microenvironment. Theranostics.

[99] Du Y., Yang Z., Sun Q., Lin M., Wang R., Peng Y., Chen X., Qi X. (2021). Engineered Microglia Potentiate the Action of Drugs against Glioma Through Extracellular Vesicles and Tunneling Nanotubes. Adv. Health Mater..

[100] Fung L.K., Ewend M.G. (1998). Pharmacokinetics of interstitial delivery of carmustine, 4-hydroperoxycyclophosphamide, and paclitaxel from a biodegradable polymer implant in the monkey brain. Cancer Res..

[101] Schinkel A.H. (1999). P-Glycoprotein, a gatekeeper in the blood-brain barrier. Adv. Drug Deliv. Rev..

